# Anesthetic management for cesarean section in a patient receiving transplacental treatment of fetal tachyarrhythmia: a case report

**DOI:** 10.1186/s40981-019-0251-0

**Published:** 2019-05-07

**Authors:** Nobuhiro Tanaka, Tomoaki Fujii, Niina Mikami, Yuka Uchinami, Hitoshi Saito, Yuji Morimoto

**Affiliations:** Department of Anesthesiology and Critical Care Medicine, Hokkaido University Graduate School of Medicine, Kita-15, Nishi-7, Kita-ku, Sapporo, 060-8638 Japan

**Keywords:** Cesarean section, ClearSight™ system, Fetal tachyarrhythmia, Non-invasive continuous hemodynamic monitoring, Transplacental anti-arrhythmic treatment

## Abstract

**Background:**

Although rare, long-lasting fetal tachyarrhythmia often leads to fetal heart failure and hydrops. Some mothers receive transplacental treatment of fetal tachyarrhythmia (TTFT), which can potentially worsen maternal hypotension and bradycardia. Moreover, the use of rescue cardiovascular agents intraoperatively can worsen fetal tachycardia. However, reports of the anesthetic management of patients receiving TTFT are rare.

**Case presentation:**

A 31-year-old woman who was receiving digoxin and sotalol for TTFT underwent planned elective cesarean section. The fetus had hypoplastic left heart syndrome, hydrops, and tachycardia. We used combined spinal-epidural anesthesia with a reduced dose of local anesthetic. We also employed a non-invasive continuous hemodynamic monitoring system. The mother’s systolic blood pressure remained at ≥ 90% of the baseline value; intraoperative administration of rescue cardiovascular agents was not required.

**Conclusions:**

We successfully anesthetized a woman for cesarean section, who was receiving TTFT for fetal tachyarrhythmia, using combined spinal-epidural anesthesia and non-invasive continuous hemodynamic monitoring.

## Background

Although fetal tachyarrhythmia is encountered in less than 0.1% of pregnancies [1], hydrops has been reported in up to 30–40% of fetuses with supraventricular tachycardia and in 7–43% of those with atrial flutter [2, 3]. The perinatal mortality associated with fetal hydrops has been reported as 35% regardless of treatment [4]. Several studies have described the efficacy of digoxin, sotalol, flecainide, and amiodarone for transplacental treatment of fetal tachyarrhythmia (TTFT) [5, 6]; however, a standard protocol has yet to be established. A prospective multicenter trial to establish a standardized protocol for TTFT is currently underway in Japan in which mothers are administered digoxin monotherapy, digoxin with sotalol, or digoxin with flecainide [4]. Hence, we believe that the combination of digoxin and sotalol used in this case will become mainstream for TTFT in the future.

TTFT does not limit the mode of delivery to vaginal delivery or cesarean section, but reports describing the anesthetic management of patients receiving TTFT are rare. A single report describes the anesthetic management of a woman undergoing a cesarean section while receiving TTFT, but high-dose amiodarone was used in that case [7]. To the best of our knowledge, the present report is the first to describe the anesthetic management of a parturient receiving digoxin and sotalol for TTFT.

In planning the anesthetic management, we were concerned that spinal anesthesia may cause maternal hypotension and bradycardia, both of which may be exacerbated by the anti-arrhythmic drugs. Additionally, the intraoperative use of rescue cardiovascular agents, such as ephedrine and atropine, could worsen fetal tachycardia. Thus, continuous hemodynamic monitoring seemed warranted in this case. Here, we describe the successful anesthetic management of cesarean section in a mother who was taking digoxin and sotalol for TTFT.

## Case presentation

A 31-year-old woman (161 cm, 59.2 kg, 48 kg before pregnancy, gravida 1, para 0, abortus 0) who was receiving digoxin and sotalol for TTFT was scheduled to undergo an elective cesarean section.

Fetal tachyarrhythmia was first identified at 26 weeks and 0 days of gestation, and the fetus was found to have hypoplastic left heart syndrome (HLHS), hydrops, and tachycardia with a heart rate (HR) of around 250 beats per minute (bpm). The mother received digoxin (0.75 mg/day) and sotalol (320 mg/day) for TTFT from 26 weeks and 1 day of gestation to the day of surgery. The fetal tachycardia and hydrops immediately improved, and the fetal HR stabilized at 140–150 bpm. The maternal HR was in the 60 s before starting TTFT and in the 50 s after starting TTFT. The maternal blood concentration of digoxin remained at 1.3–1.5 ng/mL, and she experienced no adverse events. The cesarean section was scheduled at 37 weeks and 5 days of gestation to facilitate the treatment schedule of the infant’s HLHS.

Combined spinal-epidural anesthesia (CSEA) was chosen for the cesarean section. The ClearSight™ system (Edwards Lifesciences Corp., Irvine, CA, USA) was used in addition to the standard hemodynamic monitoring. The ClearSight™ finger cuff was placed on the mother’s left middle finger, and a non-invasive blood pressure (NIBP) monitor (IntelliVue MP70; Philips Electronics Japan Corp., Tokyo, Japan) was placed on her right upper arm. Intravenous access was established using a 20 G cannula. The patient was given 400 mL of 6% hydroxyethyl starch 130/0.4/9 (Voluven®^□^, Fresenius Kabi, Bad Hamburg, Germany) from when she arrived in the operating theater until the start of the operation. CSEA was performed with the patient in the left lateral decubitus position. An epidural catheter was inserted at the T12–L1 level using an 18G Tuohy needle. Spinal anesthesia was performed at the L3–4 levels using a 27 G Quincke needle, and 0.5% hyperbaric bupivacaine 9 mg and fentanyl 15 mcg were administered intrathecally. The surgery began after confirming the loss of cold sensation up to the T8 dermatome.

We sought to maintain the mother’s systolic blood pressure (SBP) at ≥ 90% of the baseline value. If her SBP fell below 90% of the baseline, we planned to administer phenylephrine if her HR was > 60 bpm or ephedrine 4–8 mg if her HR was < 60 bpm. Her baseline blood pressure was 116/71 mmHg by the ClearSight™ system, and her SBP as measured by the ClearSight™ system stayed over 115 mmHg until the infant’s birth. The SBP value measured by the NIBP cuff was somewhat lower than that measured by the ClearSight™ system, but the difference was within 10%.

**Fig. 1:**
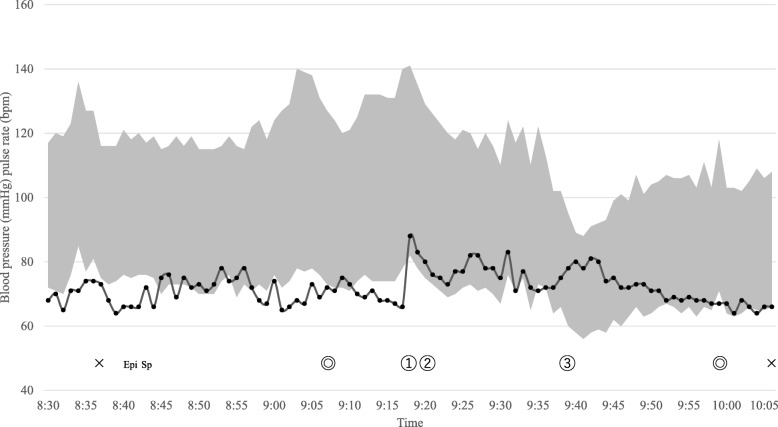
Vital sign record during cesarean section. Gray area shows blood pressure measured by the ClearSight™ system. Epi: epidural placement, Sp: spinal anesthesia induction, ●: pulse rate, × − ×: anesthesia time, ◎–◎: operation time, ①: birth of the infant, ②: administration of methylergometrine 0.2 mg, ③: administration of metoclopramide 10 mg

The duration of anesthesia was 94 min, and the duration of surgery was 52 min (Fig. 1). The total amount of fluid administered was 1055 mL, which comprised crystalloid 655 mL and colloid 400 mL. The total blood loss, including amniotic fluid, was 745 mL and the mother produced 200 mL of urine. The infant’s birth weight was 2618 g, and the Apgar scores were 8, 8, and 8 at 1, 3, and 5 min, respectively. The infant’s initial umbilical artery pH was 7.252. The fetal HR was in the 140 s before entering the operating theater, and the infant’s HR was in the 130 s upon admission to the neonatal intensive care unit. Sotalol was administrated to the infant from the first day of life to prevent tachyarrhythmia and the infant’s HR remained stable at 120–150 bpm.

The infant underwent a pulmonary artery banding operation at 4 days of age, the Norwood operation at 20 days of age, and the bidirectional Glenn operation at 3 months of age. The mother was found to have an elevated D-dimer level (21.66 μg/mL) on postoperative day (POD) 7. Lower extremity venous ultrasonography and contrast-enhanced computed tomography were performed to rule out deep vein thrombosis; however, there were no abnormal findings and she was discharged home on POD 10.

## Discussion

To the best of our knowledge, this is the first case report describing the anesthetic management for cesarean section in a parturient receiving digoxin and sotalol for TTFT. Amiodarone is the least preferred choice for TTFT due to its side effects such as neonatal hypothyroidism [8]. The first-line drug of choice for treating fetal tachycardia remains controversial, but digoxin is usually used, followed by sotalol or flecainide either alone or in combination with digoxin. In cases with fetal hydrops, the combination of digoxin and sotalol is accepted as first-line therapy [4]. Sotalol is a beta-adrenoceptor blocker that also has class III anti-arrhythmic properties. Although sotalol is less well reported in the literature, its use is increasing [5, 6].

We carefully considered the potential anesthetic risks in this case. Firstly, the bradycardia that may occur following spinal anesthesia could have been exacerbated by the anti-arrhythmic drugs the mother was taking. Although digoxin was not found to increase the risk of bradycardia after neuraxial anesthesia in a previous study, beta-adrenoceptor blockers are suggested as a risk factor of moderate bradycardia (HR of 40–50 bpm) [9]. Another study found a higher incidence of bradycardia after a neuraxial block when the analgesic level was at or above the T4 dermatome [10].

Secondly, we sought to minimize the use of atropine and ephedrine intraoperatively since these drugs may worsen fetal tachycardia. We also considered the management of maternal hypotension because phenylephrine could have caused maternal bradycardia requiring atropine treatment [11]. Moreover, ephedrine’s beta-adrenergic effect may be attenuated by the sotalol. In order to minimize maternal hypotension, bradycardia, and the need for rescue cardiovascular agents, we decided to employ a sensitive, continuous hemodynamic monitoring device in addition to standard monitoring.

The most common definitions of hypotension during cesarean section are either SBP < 80% of the baseline value or < 100 mmHg [12]; however, current guidelines recommend that SBP should be maintained at ≥ 90% of the baseline after induction of spinal anesthesia [12]. To avoid maternal and fetal adverse effects, we elected to maintain the mother’s SBP at ≥ 90% of the baseline. Similarly, we decided to use an HR cutoff of 60 bpm to decide whether to treat intraoperative hypotension with either phenylephrine or ephedrine according to a previous study [13]. Fortunately, the mother’s SBP remained above 115 mmHg and ≥ 90% of the baseline, and no rescue cardiovascular agents were required before the infant’s birth.

The relatively low analgesic level (T8) likely accounted for the mother’s stable hemodynamics. We used hyperbaric bupivacaine 9 mg, which is slightly lower than the recommended dose of 10 mg according to Harten’s dose chart [14, 15]. We were also concerned about the potential maternal bradycardia associated with the use of intrathecal opioids, such as morphine [16]. We elected to use fentanyl, which was not associated with maternal bradycardia in a previous report [17]. The mother did not complain of any pain during the surgery.

In recent years, studies have reported the use of the ClearSight™ (previously called Nexfin™) continuous non-invasive hemodynamic monitoring system during cesarean section [13, 18]. These reports demonstrated that use of the ClearSight™ system helped minimize the incidence of intraoperative maternal hypotension. In the present case, there were small differences between the blood pressure values obtained by the NIBP cuff and the ClearSight™ system. Indeed, another study found large limits of agreement between direct radial arterial pressure and the ClearSight™ system [19]. Accordingly, invasive continuous blood pressure monitoring may still be superior if either the mother or fetus is hemodynamically unstable.

## Conclusions

As care improves for infants born with congenital cardiac abnormalities, the use of TTFT will likely increase. There are several important anesthetic considerations in patients who are receiving this type of treatment, including the risk of maternal bradycardia and hypotension, as well as the potential risks of using rescue cardiovascular agents. In this case, we successfully provided a safe and effective anesthetic using CSEA and non-invasive continuous hemodynamic monitoring in a patient undergoing cesarean section who was receiving a combination of digoxin and sotalol for TTFT.

## Abbreviations

bpm: Beats per minute
CSEA: Combined spinal-epidural anesthesia
HLHS: Hypoplastic left heart syndrome
HR: Heart rate
NIBP: Non-invasive blood cuff pressure
SBP: Systolic blood pressure
TTFT: Transplacental treatment of fetal tachyarrhythmia
